# The N-Terminal Region of *Plasmodium falciparum* MSP10 Is a Target of Protective Antibodies in Malaria and Is Important for PfGAMA/PfMSP10 Interaction

**DOI:** 10.3389/fimmu.2019.02669

**Published:** 2019-11-20

**Authors:** Hikaru Nagaoka, Bernard N. Kanoi, Kana Jinoka, Masayuki Morita, Thangavelu U. Arumugam, Nirianne M. Q. Palacpac, Thomas G. Egwang, Toshihiro Horii, Takafumi Tsuboi, Eizo Takashima

**Affiliations:** ^1^Division of Malaria Research, Proteo-Science Center, Ehime University, Matsuyama, Japan; ^2^Department of Malaria Vaccine Development, Research Institute for Microbial Diseases, Osaka University, Suita, Japan; ^3^Med Biotech Laboratories, Kampala, Uganda

**Keywords:** malaria, *Plasmodium falciparum*, blood-stage vaccine, PfMSP10, PfGAMA

## Abstract

Clinical manifestation of malaria is mainly due to intra-erythrocytic development of *Plasmodium* parasites. *Plasmodium falciparum* merozoites, the invasive form of the blood-stage parasite, invade human erythrocytes in a complex but rapid process. This multi-step progression involves interactions between parasite and human host proteins. Here we show that antibodies against a vaccine antigen, PfGAMA, co-immunoprecipitate with PfMSP10. This interaction was validated as direct by surface plasmon resonance analysis. We then demonstrate that antibodies against PfMSP10 have growth inhibitory activity against cultured parasites, with the region PfMSP10 R1 that is critical for its interaction with PfGAMA being the key target. We also observe that the PfMSP10 R1 region is highly conserved among African field isolates. Lastly, we show that high levels of antibodies against PfMSP10 R1 associate with reduced risk to clinical malaria in children resident in a malaria endemic region in northern Uganda. Put together, these findings provide for the first time the functional context of the important role of PfGAMA/PfMSP10 interaction in erythrocyte invasion and unveil a novel asexual blood-stage malaria vaccine target for attenuating *P. falciparum* merozoite invasion.

## Introduction

*P. falciparum* malaria is a serious global health and economic burden, with an estimated 219 million cases and 435,000 related deaths in 2017 alone (1). Efforts geared toward malaria elimination place the development of a highly efficacious vaccine against *P. falciparum* as an urgent global agenda. Recent data suggest that an effective vaccine will need to be multi-component, targeting different antigens, or multiple stages of the parasite life cycle (2, 3). A multi-component malaria vaccine may broaden and sustain vaccine induced protective immunity, hence overcoming antigenic diversity and parasite immune evasion, both of which are major drawbacks of current vaccine candidates (4). This imperative provides a strong rationale for the identification of novel blood-stage antigens (5) for downstream vaccine development. Parasite proteins involved in the complex, tightly regulated, multistep process of host erythrocyte invasion provide unique and potentially viable vaccine targets. These proteins are mostly glycosylphosphatidylinositol (GPI) anchored on the merozoite surface or initially stored in apical organelles (i.e., micronemes, rhoptries, and dense granules) and later translocated onto the surface of the invading parasite (6, 7). For example, the blocking of merozoite surface proteins 1 and 2 (MSP1 and MSP2, respectively) and the GPI-anchored micronemal antigen (PfGAMA) inhibit parasite development (8, 9). These antigens are also targets of naturally acquired human immunity in malaria exposed populations (8, 10).

Protein-protein interactions are important drivers of host-pathogen recognition, erythrocyte invasion, and malaria pathology. One example of an essential interaction required for erythrocyte invasion is PfRipr/CyRPA/PfRh5, an adhesive complex with human basigin (11). Similarly, interaction between apical membrane antigen 1 (AMA1), which is translocated to the merozoite surface, and rhoptry neck protein 2 (RON2), which is transferred to the erythrocyte membrane during invasion, is essential for tight junction formation, an irreversible step that commits the parasite to invasion (11, 12). These interfaces of protein-protein interactions are attractive targets for vaccine induced immunity and/or small molecule inhibitors as they could allow impediments at high specificity.

Recently we identified PfGAMA as a merozoite protein essential for erythrocyte invasion (9), but its precise function in invasion is unknown. The protein, which is refractory to gene knockout, is initially stored in the microneme and subsequently translocated to the parasite surface before the merozoite invades a new erythrocyte (9). PfGAMA possesses an erythrocyte binding epitope region (13) and anti-PfGAMA antibodies inhibit *P. falciparum* merozoite invasion (9). In this study, using the wheat germ cell-free protein expression system (WGCFS) (14), we attempted to identify, synthesize, and characterize PfGAMA interacting partners that could be important for merozoite invasion of erythrocytes, and can be targeted for malaria vaccine development. We observed that PfGAMA interacts with another GPI-anchored protein, PfMSP10, and antibodies against the N-terminal region of PfMSP10 responsible for the interaction induced growth inhibition activity in cultured *P. falciparum* parasites.

## Materials and Methods

### Ethics Statement

Human sera were obtained from residents (*n* = 66) of a malaria endemic region in Northern Uganda who were enrolled and prospectively monitored for symptomatic malaria episodes for a year. The study, which has been extensively reported (10, 15), was approved by the ethical institutional review committees of Ehime University, Osaka University, Japan; Med Biotech Laboratories (IRB-00003995-MBL-BIOMEDICAL), Lacor Hospital (LHIREC 023/09/13); and the Uganda National Council for Science and Technology (HS866, HS1403).

All animal immunizations were commercially conducted at Kitayama Labes Co., Ltd. (Ina, Japan). All efforts were made to minimize animal suffering.

### Production of Recombinant Proteins and Antisera

We generated rabbit and mouse antisera as described (16) against ecto-PfGAMA (N_25_-G_715_); ecto-PfMSP10 (D_29_-Q_507_); PfMSP10 region (R) 1, amino acid D_29_-N_188_; R2, N_110_-E_268_; R3, I_189_-V_348_; R4, E_269_-R_428_; and R5, N_349_-Q_507_. Briefly, wheat codon optimized PfGAMA (Table S1) and PfMSP10 sequences were purchased from Genscript (Tokyo, Japan), cloned into the WGCFS pEU-E01-GST vector (CellFree Sciences, Matsuyama, Japan), and expressed as N-terminal glutathione S-transferase (GST) fusion proteins using WGCFS (CellFree Sciences). PfMSP10 regions were amplified from the plasmid using specific primers (Table S1). The amplified DNA was restricted with XhoI and NotI, ligated into the pEU-E01-GST vector, and expressed as N-terminal GST fusion protein using WGCFS. The proteins were affinity purified using the GST tag, separated in a 12.5% SDS-polyacrylamide gel, and stained with Coomassie brilliant blue R-250 (CBB). The recombinant proteins were used for antisera production. Additional antibodies used in this study are described elsewhere (16).

### Parasite Culture, Immunoprecipitation, and Western Blot Analysis

Parasite culture and Western blot screening was conducted as described (16, 17). For immunoprecipitated samples, Western blot analysis was performed with a panel of mouse antisera against 286 antigens (16) each at 1/1,000 dilution. To select proteins that potentially interact with PfGAMA, the following criteria were applied: (i) detection of a single major band in the anti-PfGAMA immunoprecipitated sample and (ii) the corresponding band being absent in the negative control sample which was immunoprecipitated with anti-GST antibodies. Of the 286 antigens screened, only PfMSP10 protein passed the above criteria.

### Surface Plasmon Resonance (SPR)

All SPR experiments were performed using a Biacore X100 instrument (GE Healthcare, Camarillo, CA) according to the manufacturer's instructions (16). After subtraction of the contribution of bulk refractive index, and non-specific interactions with the CM5 chip surface, individual association (*k*_a_) and dissociation (*k*_d_) rate constants were obtained by global fitting in a 1:1 binding model equation. Measurement conditions were optimized so that the contribution of mass transport to the observed *K*_D_ values was negligible.

### Growth Inhibition Assay (GIA)

Total rabbit IgGs to PfMSP10, PfMSP10 truncates, EBA175, and His-GST for GIA were purified from respective rabbit antisera with HiTrap protein G-Sepharose columns (GE Healthcare) according to the manufacturer's protocol. GIA was performed as per an established protocol (17).

### Erythrocyte Binding Assay (EBA)

EBA was performed as described (9). In brief, 90 μl of recombinant PfMSP10, PfRh5, and His-GST (1 μg) was incubated with 10 μl of packed human erythrocytes on a rotating wheel for 60 min at RT. Erythrocyte binding was subsequently measured by flow cytometry using a FACSCantII (BD Biosciences, San Jose, CA) with an acquisition of 50,000 events per sample. Data were analyzed with FlowJo 9.1 software (Tree Star, Ashley, OR). Three independent experiments were performed.

### Immunofluorescence Assays (IFA)

Free merozoites were isolated as reported (17) and fixed at room temperature (RT) for 30 min with 4% paraformaldehyde/0.0075% glutaraldehyde in PBS. The merozoites were processed with or without permeabilization with 0.1% Triton X-100, and blocked with 3% bovine serum albumin (BSA) in PBS at 37°C for 30 min. The slides were stained with rabbit anti-PfMSP10 antibody, 1:1,000; rabbit anti-PfGAMA, 1:1,000; or mouse anti-PfMTIP antibody, 1:100. Secondary antibodies, Alexa Fluor 488-conjugated goat anti-rabbit IgG, and Alexa Fluor 568-conjugated goat anti-mouse IgG (Invitrogen, Carlsbad, CA), were used at a 1:1,000 dilution at 37°C for 30 min. For nuclei staining, DAPI (4′,6-diamidino-2-phenylindole) at 2 μg/ml was also added. The merozoites were then immobilized on polyethyleneimine-coated coverslips and mounted in ProLong Gold Antifade reagent (Invitrogen). High-resolution image capture and processing were performed with confocal scanning laser microscopes (LSM710 and LSM700; Carl Zeiss MicroImaging, Thornwood, NY) using 63 × oil immersion lens. Images were processed by Image J (NIH).

### AlphaScreen

Human serum antibody levels to WGCFS-expressed PfMSP10 and the various truncated forms were quantified by AlphaScreen; a homogeneous system that detects protein interactions. A detailed protocol is reported elsewhere (10).

### Statistical Analysis

The Kruskal–Wallis test was used to test the significance of differences in EBA values between groups followed by the Bonferroni correction test. Antibody associations with age were examined by Kruskal–Wallis with the Bonferroni–Dunn *post-hoc* test. The risk of acquiring clinical malaria was assessed using the Cox proportional hazard model comparing individuals with antibodies above the population median (*n* = 33) and those below (*n* = 33). Kaplan–Meier plots were used to generate curves for time-to-first clinical episode. Analyses were performed either by GraphPad Prism (GraphPad Software, San Diego, CA) or R software (version 3.1.2; R Foundation for Statistical Computing).

## Results

### PfGAMA and PfMSP10 Interact Directly

**Figure 1:**
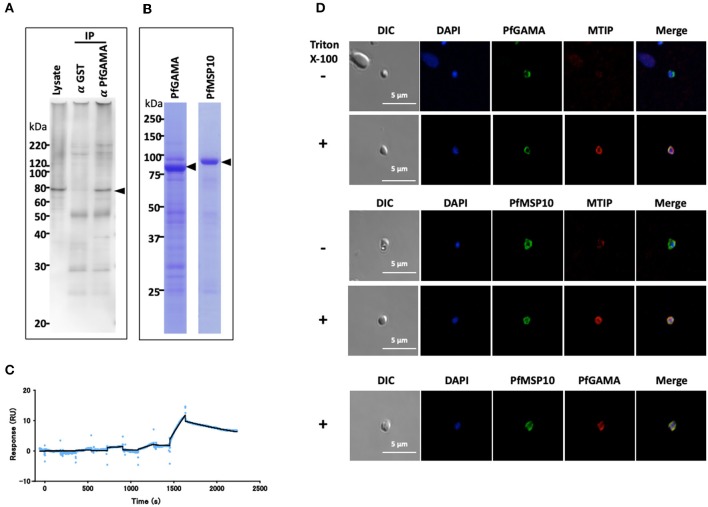
PfGAMA and PfMSP10 interact directly. **(A)** Immunoprecipitation experiment using rabbit anti-PfGAMA antibody. In Western blot analysis, mouse anti-PfMSP10 antibody detected PfMSP10 as an ~80 kDa band (arrowhead). Lysate refers to parasite lysate derived from a 10^8^ trophozoite-/schizont-rich parasite pellet; α GST IP, sample immunoprecipitated with rabbit anti-His-GST antibody as a negative control; α GAMA IP, sample immunoprecipitated with anti-PfGAMA antibody. **(B)** Purified GST-tagged recombinant PfGAMA and PfMSP10 separated by 12.5% SDS-PAGE and stained with CBB. Arrowheads indicate molecular masses predicted from amino acid sequences. **(C)** SPR single-cycle kinetics analyses sensorgrams. Purified His-tagged recombinant PfGAMA was used as ligand and PfMSP10 as analyte at increasing concentrations of 0.16, 0.8, 4, 20, and 100 nM. Blue dots indicate experimental data while black lines indicate line of fit used to calculate the kinetic parameters. **(D)** IFA analysis of PfGAMA and PfMSP10. As labeled, free merozoites were co-stained with anti-PfGAMA antibodies, anti-PfMTIP (myosin tail interacting protein) antibodies, or anti-MSP10 antibodies, with (+) and without (–) permeabilization with 0.1% Triton X-100. Cells were co-stained with DAPI (for parasite nuclei localization). The left most panels and right most panels shows images of differential interference contrast (DIC) and merged pictures, respectively.

*P. falciparum* utilizes several self and host proteins to recognize and invade erythrocytes (18). Here, we set to investigate how the parasite-encoded protein PfGAMA is involved in erythrocyte invasion (9). We hypothesized that PfGAMA interacts with multiple parasite proteins to drive the invasion process. Since immunoprecipitation assay is a powerful tool for comprehensive analysis of protein-protein interactions in parasite cultures (16, 19), we used it to identify potential PfGAMA interacting partners. Specifically, we immunoprecipitated PfGAMA protein in Nonidet P-40 (NP40; Nacalai Tesque, Inc.) solubilized schizont-rich parasites samples using rabbit anti-PfGAMA antibodies. By Western blot assay the immunoprecipitate was probed with a panel of mouse antibodies raised against 286 individual *P. falciparum* recombinant proteins (16). We observed that anti-PfGAMA antibody could co-immunoprecipitate PfMSP10 (PlasmoDB ID: PF3D7_0620400) (Figure 1A, arrowhead). By comparison, anti-GST antibodies did not immunoprecipitate PfMSP10.

To characterize the interaction in detail, purified recombinant GST-tagged PfGAMA and GST-tagged PfMSP10 (which resolved around 85 and 90 kDa, respectively; Figure 1B) were subjected to surface plasmon resonance (SPR) analysis. In good agreement with the pulldown data, PfGAMA and PfMSP10 directly interacted with a *K*_D_ value of 1.0 × 10^−7^ M (Figure 1C), suggesting that the two proteins form a complex. Negative control His-GST recombinant protein did not show specific interactions with GST-tagged PfGAMA (Figure S1). Other parameters determined by the SPR are shown in Table S2.

Using IFA, we observed that both PfGAMA and PfMSP10 co-localized on the surface of free merozoites (Figure 1D). This is consistent with a report suggesting surface localization of the two proteins (9, 20) for invasion-related functions. In addition, we previously observed that PfGAMA has a role in erythrocyte invasion (9), hence we hypothesized that PfMSP10 is also involved in parasite invasion of erythrocyte.

### Anti-PfMSP10 Antibodies Have Growth Inhibition Assay (GIA) Activity

**Figure 2:**
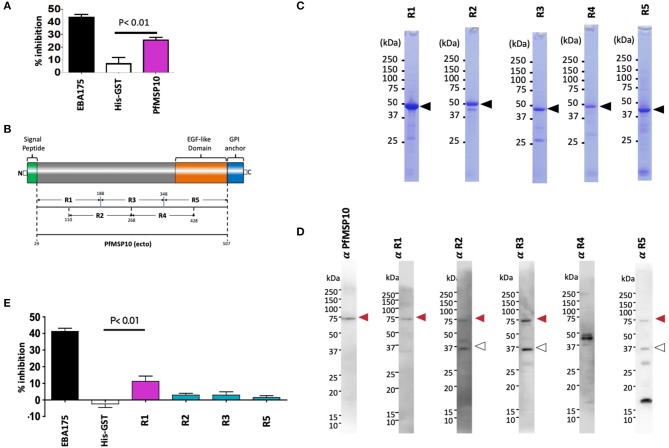
Anti-PfMSP10 antibodies have GIA activity. **(A)** GIA activity of 20 mg/ml rabbit anti-ecto-PfMSP10 antibodies. Rabbit anti-His-GST antibodies, and rabbit anti-EBA175 III-V antibodies were included as negative and positive controls, respectively. Bars indicate mean of three independent experiments, each performed in triplicate. Error bar represents the standard error of the mean. Statistically significant (student's *t*-test; *p* < 0.01) data relative to rabbit antibodies against His-GST is shown. **(B)** Schematic representation of ecto-PfMSP10 and 5 recombinant fragments that were synthesized. Numbers represent amino acid positions. The protein has a predicted N-terminal signal peptide (1–28 aa), an EGF like domain (shown in orange), and a C-terminal GPI anchor. Recombinant PfMSP10 truncates/regions were expressed as N-terminal GST-tagged proteins by the wheat germ cell-free system included region (R)1, amino acid D_29_-N_188_; R2, N_110_-E_268_; R3, I_189_-V_348_; R4, E_269_-R_428_; and R5, N_349_-Q_507_. **(C)** Purified recombinant GST-tagged PfMSP10 regions separated by 12.5% SDS-PAGE and stained with CBB. Arrowheads indicate molecular masses as predicted from amino acid sequences. **(D)** Reactivity of rabbit anti-PfMSP10 antibodies to native PfMSP10. Schizont-rich parasite pellet lysate was resolved under reducing conditions and probed with rabbit antibodies specific to each region. The additional bands (open arrowheads) represent processed fragments. **(E)** GIA activity of 20 mg/ml rabbit anti-PfMSP10 antibodies raised against each region. Rabbit anti-His-GST antibodies, and rabbit anti-EBA175 III-V antibodies were included as negative and positive controls, respectively. Bars indicate mean of two independent experiments, each performed in triplicate. Error bar represents standard error of the mean. Statistically significant (student's *t*-test; *p* < 0.01) data relative to rabbit antibodies against His-GST is shown.

To evaluate if PfMSP10 is important for *P. falciparum* development, we tested the potency of anti-PfMSP10 antibodies to block parasite growth by *in vitro* GIA. Rabbit anti-PfMSP10 antibodies (20 mg/ml) inhibited growth of the 3D7 parasite strain by 26 ± 2% (mean ± SE), significantly higher than that of the negative control (anti-His-GST antibodies, *p* < 0.01; Figure 2A). Rabbit antibodies to EBA175 (region III-V; positive control) and His-GST (negative control) inhibited growth by 44 ± 2 and 7 ± 4% (mean ± SE), respectively. These data suggest that PfMSP10 is functionally important for parasite erythrocyte invasion or development.

To determine the PfMSP10 region important for erythrocyte invasion and the key target of antibodies with GIA activity, we synthesized five approximately equally sized and overlapping truncates of PfMSP10 as GST-fused proteins (Figures 2B,C). Consistent with published data (20), antibodies raised against each truncate specifically recognized parasite native PfMSP10 at the expected ~75 kDa for full length (red arrow; Figure 2D), and a ~36 kDa processed fragment (white arrow; Figure 2D). However, anti-PfMSP10 R4 antibodies did not recognize the full-length protein.

We subsequently measured the GIA activity of antibodies to each of the PfMSP10 truncates. Antibodies to PfMSP10 R1 (D_29_-N_188_**)** induced a GIA activity of 11 ± 3% which was significantly higher than that of the negative control, anti-His-GST antibodies (*p* < 0.01; Figure 2E), as well as other truncates. GIA activity of anti-PfMSP10 R4 antibodies was not tested since the antibody specific reactivity could not be confirmed by Western blot analysis (Figure 2D).

### PfMSP10 R1 (D_29_-N_188_) Is Relatively Conserved Among the *P. falciparum* Field Isolates and Is the Key Target of GIA Antibodies

**Figure 3:**
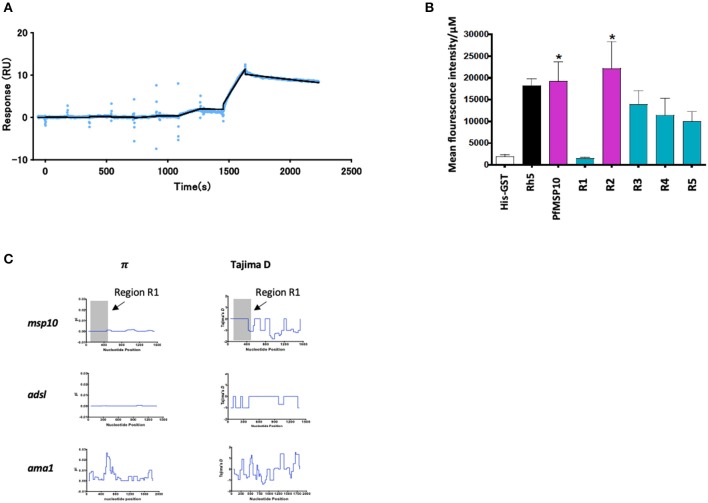
**(A)** SPR single-cycle kinetics analyses sensorgrams. Purified His-tagged recombinant PfGAMA was used as ligand and PfMSP10 R1 as analyte at increasing concentrations of 0.16, 0.8, 4, 20, and 100 nM. Blue dots indicate experimental data while black lines indicate line of fit used to calculate the kinetic parameters. **(B)** Ecto-PfMSP10 and PfMSP10 regions binding to erythrocyte surface were quantified by FACS and presented as mean fluorescence intensity. PfRh5 and His-GST were included as positive and negative controls, respectively. **(C)** Sliding window analysis showing nucleotide diversity (π) values and Tajima's D statistic in *msp170*. The gray box represents Region 1 of amino acid residues 29–188. Also analyzed as controls were *Adsl*, a highly conserved housekeeping gene; and *ama1*, a highly polymorphic protein.

We further sought to understand the potential mode of action of the antibodies against PfMSP10 R1. We hypothesized that PfMSP10 R1 interacts with PfGAMA and/or erythrocyte surface proteins. First, to clarify if PfMSP10 R1 binds directly to PfGAMA, we analyzed their interaction by SPR. Kinetics analysis showed direct interaction with *K*_*D*_ = 6.50 × 10^−7^ M (Figure 3A), suggesting that PfMSP10 R1 is responsible for the interaction with PfGAMA. Other truncates/regions did not show detectable interactions (data not shown).

Secondly, we sought to investigate if PfMSP10 interacts with the erythrocyte surface. Using ecto-PfMSP10 as well as the five regions, we performed erythrocyte binding assays (EBA). We observed that recombinant ecto-PfMSP10 as well as PfMSP10 R2 bound to the erythrocyte surface (Figure 3B). The erythrocyte binding activities of both PfMSP10 proteins were comparable to that of PfRh5, which is known to interact with basigin, an erythrocyte surface protein (21). In contrast, PfMSP10 R1 did not bind to the erythrocyte surface (*P* > 0.05), suggesting that the region essential for invasion of erythrocyte is different from the region involved in erythrocyte binding.

Thirdly, we sought to determine the genetic polymorphism of this gene in malaria endemic populations. Based on the *msp10* sequences of field isolates from different sites in West and East Africa (*n* = 110) deposited in PlasmoDB (http://plasmodb.org/), we calculated nucleotide diversity (π) and Tajima's D, indicative of diversifying selection. While the full length *pfmsp10* is moderately conserved, both metrics did not show any peaks in nucleotide sequence diversity suggesting an absence of balancing selection of R1 (Figure 3C, gray area). These were compared with the controls, *adsl*, a highly conserved housekeeping adenylosuccinate lyase gene, and *ama1*, which encodes the highly polymorphic apical membrane antigen 1 (AMA1) protein.

These results suggest that the N-terminal region of PfMSP10 is important for erythrocyte invasion and development.

### Individuals With Anti-PfMSP10 R1 Antibodies Have Reduced Risk of Clinical Malaria

**Figure 4:**
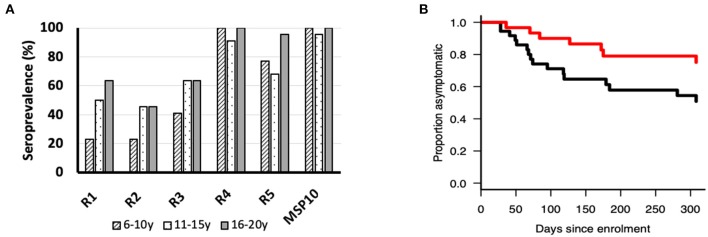
**(A)** Seroprevalence (%) of ecto-PfMSP10 and PfMSP10 regions in different age groups; 6–10 years (*n* = 22), 11–16 years (*n* = 22), and 16–20 years (*n* = 22). All PfMSP10 regions were immunogenic in natural infections. **(B)** Kaplan–Meier plot of the probability of remaining free from clinical malaria during the 1-year follow-up in respect to antibodies against PfMSP10 R1. Individuals with high antibody levels, above population median (red), are compared to those with low levels (black) (*p* < 0.05).

Since anti-PfMSP10 antibodies inhibited merozoite invasion *in vitro*, and we recently observed that PfMSP10 is recognized by sera from African children (10), we sought to investigate whether PfMSP10 regions are differentially recognized by human sera samples from a malaria endemic area. All PfMSP10 regions were well-recognized by participants in different age groups (Figure 4A). For all PfMSP10 regions, the mean antibody levels for the cohort were higher than for malaria-naive individuals, reflecting the specificity of *P. falciparum* antibody responses (data not shown). The breadth of antibody responses appeared to differ according to PfMSP10 region; specifically, regions in close proximity to the less conserved C-terminal (R4 and 5) had higher seropositivity (>70%) than the relatively more conserved N-terminal R1 and R2 regions which had seropositivity ranging between 22 and 63% in different age groups (Figure 4A). Consistent with other protective antigens (10, 22), the intensity of antibody responses to regions R1, R4, and R5 seemed to be acquired in an age-dependent, albeit non-significant, manner (Figure S3). Further analysis revealed that individuals with anti-PfMSP10 R1 antibody levels above the population median had a significantly reduced risk [unadjusted hazard ratio (HR), 0.59 (95% confidence interval; CI, 0.01–0.83); *p* = 0.047] of febrile malaria (defined as fever and ≥5,000 parasites/μl of blood) (Figure 4B). However, this association became non-significant after inclusion of potential confounding effects in the model. Put together, a short, conserved but naturally immunogenic region of PfMSP10, R1 (MSP10 D_29_-N_188_), corresponding to the PfGAMA binding region, is a key target of anti-PfMSP10 antibodies with GIA activity, and protective antibodies in natural *P. falciparum* infection, making it an ideal target for malaria vaccine studies.

## Discussion

Invasion of human erythrocytes by *P. falciparum* merozoites is an intricate and rapid process that is mediated by numerous parasite and host proteins (6, 18, 23). This multi-step progression starts with merozoite attachment to the erythrocyte surface, reorientation of the apical end to bring it into direct contact with the erythrocyte surface, followed by formation of a tight junction with the erythrocyte. These events allow parasite ligands released from merozoite apical organelles to interact with each other and with host cell receptors (6, 24). A clear understanding of the molecular interactions leading to, and during the invasion process presents a critical element for development of efficacious antimalarial interventions. This study reports the interaction between the proteins PfGAMA and PfMSP10, which is important for successful merozoite invasion of erythrocytes.

Several studies suggest that immune responses targeting merozoite surface antigens contribute to naturally acquired protective immunity, and accordingly, several highly validated surface antigens have been recommended or are under consideration as asexual blood-stage malaria vaccine candidates (8, 9). Recently, we identified PfGAMA for further evaluation as a blood-stage vaccine candidate antigen (9). PfGAMA is an 85 kDa micronemal GPI-anchored protein that migrates to the surface of merozoites upon erythrocyte egress (9, 13). The protein undergoes primary and secondary processing events generating two forms of PfGAMA heterodimers: p37+p49 and p37+p42 (13). PfGAMA binds to the erythrocyte surface, with antibodies against the molecule inducing robust GIA activity (9). The PfGAMA interacting partner described here, PfMSP10, has two EGF-like domains and a GPI-anchor at the C-terminus, is expressed late in intra-erythrocytic stages, and is subjected to proteolytic processing to form a 36 kDa product (20). PfMSP10 localizes to the apical end and on the surface of merozoites in schizonts (25), and only on the surface in free merozoites (20).

In the present study, we identified for the first time direct molecular interaction between PfGAMA and PfMSP10 (*K*_D_ = 1.0 × 10^−7^ M). More specifically, the *K*_D_ value of the N-terminal PfGAMA/PfMSP10 R1 was comparable to that of ecto-PfMSP10 (*K*_D_ = 6.5 × 10^−7^ M), suggesting that PfMSP10 R1 is the key interacting region. Consistently, IFA analysis of PfMSP10 R1 and PfGAMA validated that both proteins co-localize on the surface of free merozoites. Like other GPI anchored proteins, the PfGAMA/PfMSP10 interaction could be enriched by lipid raft-like membrane on the merozoite surface (26). Although the molecular function of this complex has not been explored, we hypothesize that it is functionally important for erythrocyte invasion via recognition and binding to a yet unknown erythrocyte receptor. Alternatively, the two proteins recognize closely associated but different receptors; this interaction would then promote a receptor heterodimerization important for signal transduction in erythrocytes (27). Further investigations would be required to test this hypothesis.

**Figure 5:**
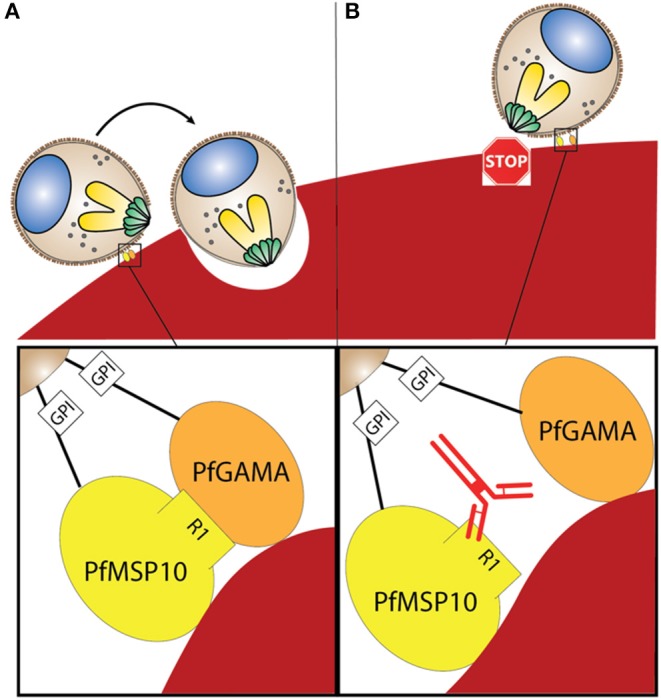
**(A)** Interaction between PfGAMA and PfMSP10 R1 is needed for erythrocyte invasion. Lipid raft-like membrane on the surface of merozoites would promote the interaction between the two GPI anchored proteins. **(B)** Blocking interaction between PfGAMA and PfMSP10 R1 with antibodies against PfMSP10 R1 impedes erythrocyte invasion.

When PfMSP10 was truncated, we observed that R2, R3, R4, and R5 had erythrocyte binding activity (Figure 3B). This was in agreement with a study where 3 peptides spanning amino acids (aa) 201-220, 221-240, and 421-440 had erythrocyte binding activities (28). Another recent study reported that human monoclonal antibodies against the PfMSP10 EGF-like domains exhibit a 40–60% *in vitro* GIA activity, albeit at a very high concentration for monoclonal antibodies (10 mg/ml) (25). In our study, antibodies to PfMSP10 R5, which contains the two EGF-like domains, did not show any GIA activity (Figure 2E). Antibodies against PfMSP10 R4, which has a partial EGF-like domain, did not recognize either full length PfMSP10 or the 36 kDa fragment (Figure 2D), and thus we could not further evaluate the biological activity of the antibody, despite the region exhibiting mild erythrocyte binding activity. The fact that PfMSP10 R1 did not show any erythrocyte binding activity despite direct interaction with PfGAMA and anti-PfMSP10 R1 antibodies having moderate invasion blocking activity, strongly suggests that the PfGAMA/PfMSP10 R1 interaction is important for erythrocyte invasion (Figure 5). However, the GIA activity observed was relatively low as compared with full length MSP10 as well as that of leading blood stage vaccine candidates such as EBA175 (used as positive control in Figures 2A,E). Therefore, for further studies, the MSP10 R1 region should be targeted not as a single antigen but as an important part of a multi-component merozoite invasion machinery.

Consistent with previous studies (20), we observed that PfMSP10 is highly immunogenic in malaria natural infection (Figure 4A). We further observed that individuals with high antibody responses against PfMSP10 R1 had reduced risk of having a clinical malaria episode (Figure 4B). This could indicate that the R1 region of PfMSP10 is a potential target of antibody mediated protective immunity against malaria. Although antibody responses to EGF-like domains (R4 and R5) were more seroprevalent when compared to other segments of PfMSP10 (Figure 4A), there was no detectable correlation with protective immunity (Figure S4). This lack of significant association may be due to the limited number of serum samples analyzed; however, it could also suggest that the robust immunogenicity targeting the EGF-like domain serves as a smoke screen for the parasite to evade host immunity [review (29)].

Like other known merozoite surface proteins, PfMSP10 may be a target of subtilisin-like serine protease (SUB1) mediated proteolytic processing to produce the final functional fragments (30, 31). Based on the PfMSP10 amino acid sequence, six SUB1 recognition sites were predicted (Figure S2), and the prediction could map the additional bands observed when parasite lysate was blotted and probed with antibodies against R2, R3, and R5; an indicator that PfMSP10 may indeed be a substrate of SUB1. Further biochemical analysis would be required to confirm these findings.

One key limitation with this study was that we could not confirm the strain transcending efficacy of antibodies against PfMSP10. In addition, relatively low GIA activity was noted. The use of a limited number of samples for evaluating naturally acquired immunity could also have missed or overestimated the significance of the immune associations hence preventing a clear generalized statement. However, we observed that the *msp10* gene, especially R1, is conserved among field isolates and is comparable to *adsl*, a highly conserved housekeeping gene (Figure 3C).

In conclusion, targeting relatively small parts of a protein presents an opportunity to develop an optimal vaccine against malaria. These interacting regions as in the case of PfGAMA/PfMSP10 R1 are more likely conserved to preserve critical protein functions. The data presented here demonstrate that PfMSP10 R1 (PfMSP10 D_29_-N_188_), a short, conserved and naturally immunogenic region corresponding to the PfGAMA binding region, as an important component of the merozoite invasion machinery. Although additional supportive data will be required, based on this study, the region warrants further evaluation for possible inclusion in multi-component blood-stage malaria vaccine strategies.

## Data Availability Statement

The datasets generated for this study are available on request to the corresponding author.

## Ethics Statement

The studies involving human participants were reviewed and approved by Institutional Review Committees of Ehime University and Institutional Review Committees of Osaka, University, Japan. In Uganda, the studies were reviewed and approved by Med Biotech Laboratories (IRB-00003995-MBL-BIOMEDICAL), Lacor Hospital (LHIREC 023/09/13), and Uganda National Council for Science and Technology (HS866, HS1403). Written informed consent to participate in this study was provided by the participants' legal guardian/next of kin. The animal study was reviewed and approved by Kitayama Labes Co., Ltd. (Ina, Japan). All animal immunizations were commercially conducted at Kitayama Labes Co., Ltd. (Ina, Japan).

## Author Contributions

TT and ET conceived and designed experiments. HN, BK, KJ, MM, TA, NP, TE, and TH conducted experiments. HN, BK, TT, and ET analyzed the data. BK, NP, TT, and ET wrote the manuscript. All authors discussed and edited the manuscript.

### Conflict of Interest

The authors declare that the research was conducted in the absence of any commercial or financial relationships that could be construed as a potential conflict of interest.
