# Supplementary material for: The N-Terminal Region of Plasmodium falciparum MSP10 Is a Target of Protective Antibodies in Malaria and Is Important for PfGAMA/PfMSP10 Interaction
Source: Front Immunol. 2019 Nov 20;10:2669. doi: 10.3389/fimmu.2019.02669 (PMC6880778; doi:10.3389/fimmu.2019.02669)
Supplement: Supplementary file 1 [file Data_Sheet_1.PDF]

Supplementary Table and Figures

The N-terminal region of *Plasmodium falciparum* MSP10 is a target of protective antibodies in malaria and is important for proteins PfGAMA/PfMSP10 interaction

Hikaru Nagaoka[1], Bernard N. Kanoi[1], Kana Jinoka[1], Masayuki Morita[1], Thangavelu U Arumugam[1#], Nirianne M. Q. Palacpac[2], Thomas G. Egwang[3], Toshihiro Horii[2], Takafumi Tsuboi[1], Eizo Takashima[1,*]

[1]Division of Malaria Research, Proteo-Science Center, Ehime University, 3 Bunkyo-cho, Matsuyama, Ehime 790-8577, Japan
[2]Department of Molecular Protozoology, Research Institute for Microbial Diseases, Osaka University, Suita, 565-0871, Japan,
[3]Med Biotech Laboratories, P.O. Box 9364, Plot 4-6 Bell Close, Port Bell Road Luzira, Kampala, Uganda

[#] Current address: Department of Biotechnology, Faculty Center for Agricultural Education and Research, Ramakrishna Mission Vivekananda Educational and Research Institute, Coimbatore 641020, TN, India

*** Correspondence:**
Eizo Takashima
Email: takashima.eizo.mz@ehime-u.ac.jp
Tel.: +81-89-927-9939

Table S1: Sequence and Primers used in this study.

Wheat codon optimized ecto-PfGAMA sequence

ATGAACAGCAACACCCCACAGGCTTTCCTCATCCCAGAGCTCAACAACAACGAGAAGAACGAGTTCAACAACAACGAGAAGAACGAGATGAACAACAACCTCAACA
ACGAGTTCAACAACAACGAGGAGAACTGCGACATCCAAAAGATCGCCGAGGAGATGATGGAGAACCTCCTGAACGAGAACGATATGTACACCAACATCATGCTGTC
GCTCCAGAACAGGCTGTCAGACGATTACCTCTGCTCTGAGCCGAAGTACGAGAACATCTGCATCCACGAGAAGGACAAGATCTCGCTCTCATTCCCATGCTCCAAC
CCGAAGTACGAGAAGCTGATCCATAAGTTCACCTTCAAGAAGCTCTGCAACTCCAAGGCCGCGTTCAACAACACCCTCCTGAAGAGCTTCATCGAGGAGGACGAGG
AGCAGAACACCTTCTCACTGATGCTCAAGCAATTCAAGATCCTCCTGACCTGCGTGGACGATGAGCTGAAGGACATCTACAAGGAGTCCATCGACCTCCTGGTCGA
TCTCAAGACCAGCATCACGGAGCTGACCCAAAAGCTCTGGTCGGGCAAGATGGTTAACGTGCTGAAGAAGAGGGAGTTCCTCATCACCGGCATCCTGTGCGAGCTC
CGCAACGGGAACAAGTCCCCACTGATCTCTAAGTCCCTGGAGTTCGAGAACCTGGGCATCCTCAAGATGAACAACGAGGAGCTCCTGAACGAGGCCTACAACGCGT
TCAGCGACTACTACTACTTCTTCCCGTACTTCATCCAAAAGCTCCTGGAGAAGGGCGGGATGATCGAGCGGCTGATCAAGATCCACGAGAACCTCACGAAGTACAG
AACCAAGGACATGGTCAACAAGATCAACGCCCAGTCCAAGGGCGAGGTTCTCAACAACGAGGATATCCTGAACAAGCTCAACGCTTACAAGCACTACACGAAGCAT
GGGGCCACCTCATTCATCCAGTCTCGGGAGGTGAAGATCGTCAACCAAAACGTCAACAACGACGATACCACGAAGAACCAACAGCAAAACGTTAACAACAACGAGA
AGCTGAACAACAACAACAACAACAACAACAACCAGCAAGTGAACAACAACAACAACAACAACAACCAACAAGTTAACAACAACAACAACAACAACAACAACCAGGT
CAACAACAACAACAACAACAACAACAACCAAGTCAACAACAACAACTATAACAACAACAACCAGGTAAACAACAACAACAACAACAACCAACAGGTGAACAACAAC
AACAACTACAACAACCAACTGAACAACAACAACTTCAACAACAACCTCCAGGTCAACAAGAACGACAAGCACGTTCCAAAGAACAACCACACCACGGCTACCCATA
CGAACAACATCCTGTACAACCCGCTCTACTCCATCAACCCCGAGAAGCCTAAGGACATCATCAAGCTCCTGAAGGATCTGATCAAGTACCTCCACATCGTGAAGTT
CGAGAACAACGAGCCCACCACGAACATCGACGAGGAGGGCATCAGGAAGCTCCTGGAGAACTCTTTCTTCGATCTGAACGACGATATCCTCATCGTCCGCCTCCTG
CTCAAGCCTCAGACGGTTATCCTGACCGTGATCCAATCCTTCATGCTCATGACCCCAAGCCCGTCGCGCGACGCTAAGGCCTACTGCAAGAAGGCGCTGATCAACG
ACCAACTCGTGCCCACGAACGATACCAACATCCTGTCAGAGGAGAACGAGCTCGTGAACAACTTCTCTACCAAGTATGTGCTGATCTACGAGAAGATGAAGCTGCA
GGAGCTCAAGGAGATGGAGGAGAGCAAGCTCAAGATGAAGTACTCCAAGACCAACCTGAGCGCGCTCCAGGTGACGAACCCACAAAACAACAAGGACAAGAACGAT
GCTTCGAACAAGAACAACAACCCCAACAACTCCAGCACGCCTCTGATCGCCGTGGTCACCGACCTCTCGGGCGAGAAGACGGAGGACATCATCAACAACAACGTCG
ATATCGCGACCCTCTCAGTCGGGGTTCAGAACACCTTCCAAGGCCCAAACGCGAAGGCTGGGCACCATCACCATCACCATTGA

Primer list

PfMSP10	Forward primers	Reverse primers
R1	ctcgagATGGATGATATAAAAAATACATCCCAGAAGAAAATTA	gcggccgcCTAATGGTGATGGTGATGGTGATTATTATATGATTCTGAATTCATGATAGATGAATTTT
R2	ctcgagATGAACTATTTAATAAGAAAAAGAAAAGATAATACTCA	gcggccgcCTAATGGTGATGGTGATGGTGTTCTATCAAATATGATTTTAGATTGTTACCCA
R3	ctcgagATGATTATCAATTCTAATGAACATAATGAAGAACAAATA	gcggccgcCTAATGGTGATGGTGATGGTGAACTAAAGGGTGTTGAGGTTGT
R4	ctcgagATGGAAAATGATGTTTCTCAAAAAAAAACTGAT	gcggccgcCTAATGGTGATGGTGATGGTGACGTGAATTGGGTCCACAT
R5	ctcgagATGAATAGTGGGGATCAATTACAACATC	gcggccgcCTAATGGTGATGGTGATGGTGTTGTTTTGTTTGAGAATTCTTTACACAAA
Ecto-PfMSP10	ctcgagATGGATGATATAAAAAATACATCCCAGAAGAAAATTA	gcggccgcCTAATGGTGATGGTGATGGTGTTGTTTTGTTTGAGAATTCTTTACACAAA

Table S2

Table S2: Kinetic constants derived from fitting SPR sensorgrams.

	ka (1/Ms)	kd (1/s)	KD (M)	Rmax (RU)	Chi^2 (RU^2)
**PfMSP10**	1.501E+4	1.497E-3	9.973E-8	73.21	0.259
**R1**	547.7	3.549E-4	6.479E-7	888.0	0.230
**His-GST**	ND	ND	ND	ND	19.3

ND; Not-determined

Fig S1

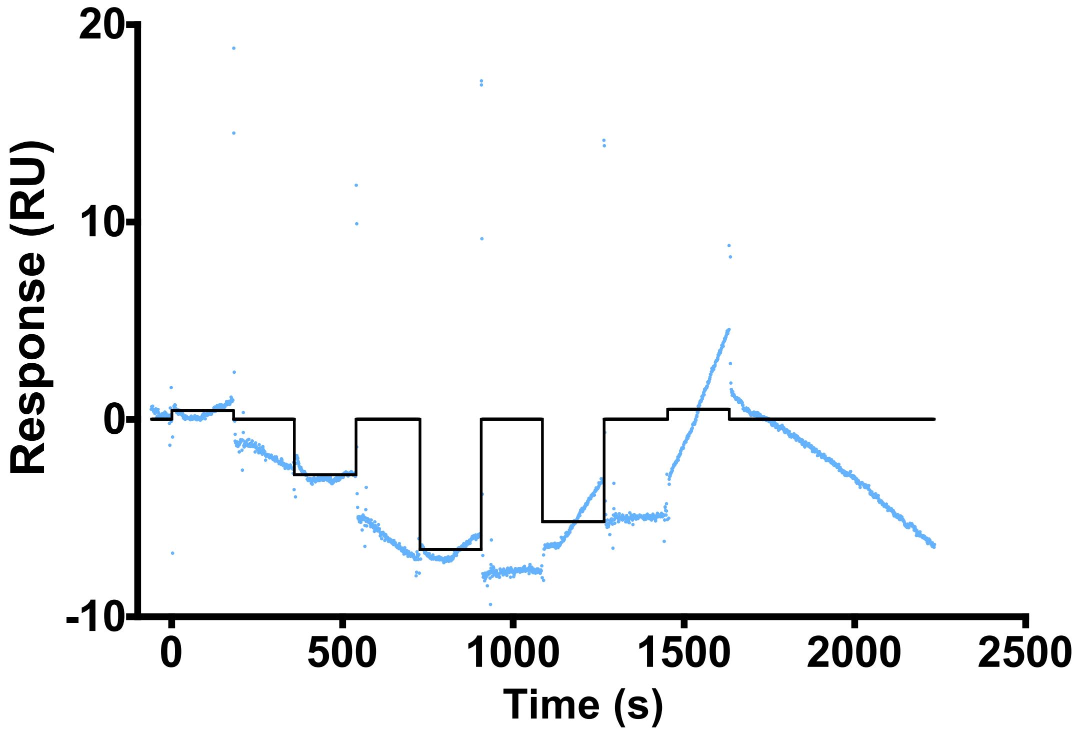

Fig S1. SPR single-cycle kinetics analyses sensorgrams for negative control (His-GST). Purified His-GST as analyte was assessed at increasing concentrations of 0.16, 0.8, 4, 20, and 100 nM. Blue dots indicate experimental data while black lines indicate line of fit used to calculate the kinetic parameters.

Fig S2

a) MMFFKCNQVFTLVFLLLLYFNNIVYTHVDDIKNTSQKKITYDKYNKNKENMNNEKNDNKDNKDNIYNDNINNDNINNDNINNEDEYKF
LSMKHYKDSLSNKLNNENDHMNYLIRKRKDNTQGSQHFNENIENNENVENNENIENNENNENIENIENNENNENNENIENNENNENNE
NSSIMNSESYNNIINSNEHNEEQIKKKEEDLIEAFFPFILKKLDNESLSLDNKYDDYYNLPNDHNDTHKENSSDHNLLGYKLGNNLKS
YLIEENDVSQKKTDDINESASSDSENIQEILSTDSNTSHLKERKNQKAPPGEHKPEVKNALLNSQVASPKGEDEKKSQPQHPLVNSGD
QLQHPKEIDENAEKIRRTLLKESRDIKNTTAIIDETVYKFEQLIMKGRYYATAVRNFVIFKVNYICEYSKCGPNSRCYIVEKDKEQCR
CRPNYIVDMSVNYFKCIPMKDMNCSKNNGGCDVNAECTIVEGAVKCQCSHLYFGDGVFCVKNSQTKQTLYILFIVILLVFQNFFI

Double Under bar = Predicted Sub1 sites
Under bar = TM and GPI-anchoring site

b)

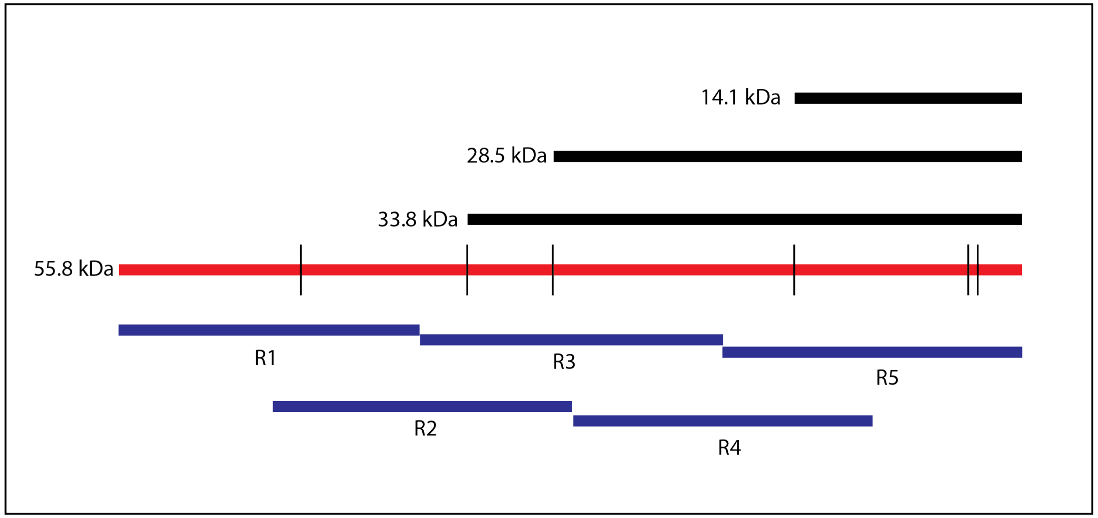

Fig S2: Six SUB1 recognition sites in PfMSP10. A previous study [1] annotation (Ile/Leu/Val/Thr-Xaa-Gly/Ala-Paa (not Leu)) was used for prediction of SUB1 sites on PfMSP10 amino acid sequence. a) The predicted SUB1 sites are highlighted in PfMSP10 amino acid sequence. b) Schematic presentation of the predicted PfSUB1 cleavage sites in PfMSP10 (vertical lines). Blue lines present the position of the truncates indicated. Black horizontal lines indicate predicted products due to the partial digestion of PfMSP10.

1. Withers-Martinez, Chrislaine, et al. "Plasmodium subtilisin-like protease 1 (SUB1): insights into the active-site structure, specificity and function of a pan-malaria drug target." *International journal for parasitology* 42.6 (2012): 597-612.

Fig S3

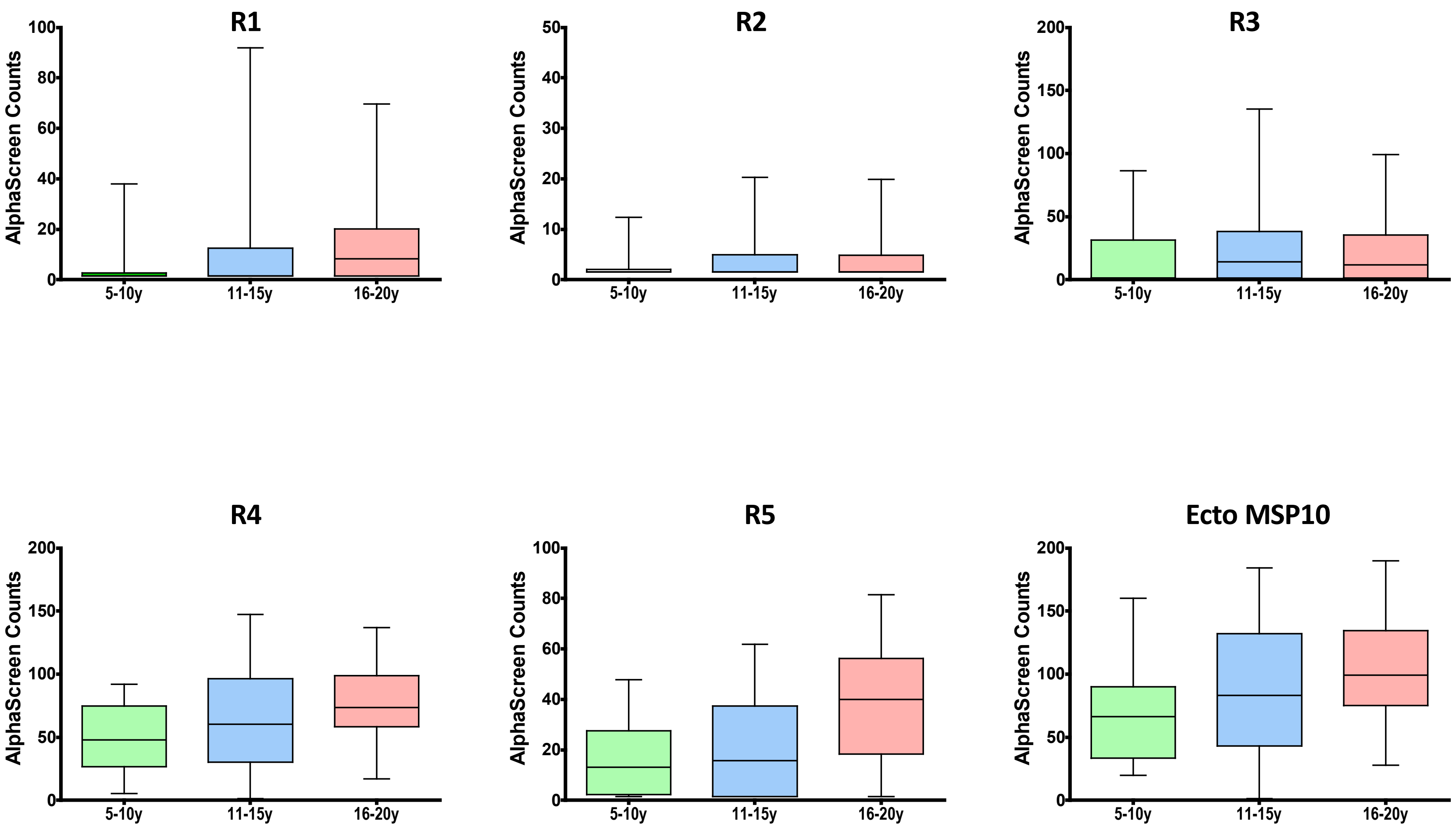

Fig S3. Distribution of antibody titres with age. A positive correlation between antibody titres to each of the PfMSP10 regions with age was observed but non was significant (Kruskal-Wallis Test P>.05).The panels show distribution of antibody titres with age for the respective antigens; A) PfMSP10 R1, B) PfMSP10 R2, C) PfMSP10 R3, D) PfMSP10 R4, E) PfMSP10 R5, and F) ecto- PfMSP10

Fig S4

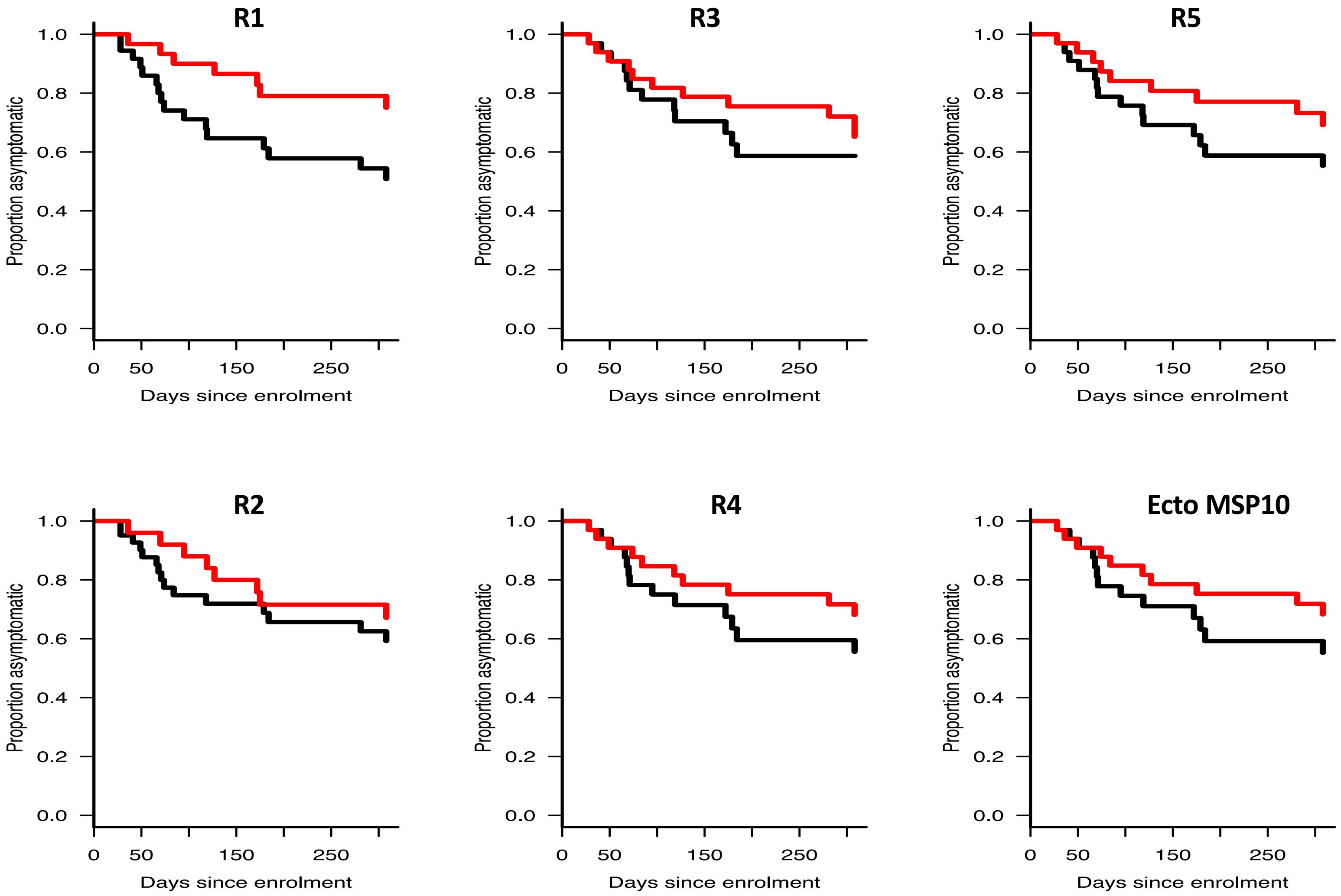

Fig S4. Kaplan–Meier plots of the probability of remaining free from clinical malaria for different regions of MSP10
